# Vascular complications after transcatheter aortic valve implantation (TAVI): risk and long-term results

**DOI:** 10.1007/s11239-013-0996-7

**Published:** 2013-10-17

**Authors:** Katarzyna Czerwińska-Jelonkiewicz, Ilona Michałowska, Adam Witkowski, Maciej Dąbrowski, Ewa Księżycka-Majczyńska, Zbigniew Chmielak, Krzysztof Kuśmierski, Tomasz Hryniewiecki, Marcin Demkow, Janina Stępińska

**Affiliations:** 1 Department of Acquired Valvular Disease, Institute of Cardiology, Alpejska 42 Street, 04-628 Warsaw, Poland; 2 Department of Radiology, Institute of Cardiology, Alpejska 42 Street, 04-628 Warsaw, Poland; 3 Department of Interventional Cardiology and Angiology, Institute of Cardiology, Alpejska 42 Street, 04-628 Warsaw, Poland; 4 Department of Cardiac Surgery and Transplantology, Institute of Cardiology, Alpejska 42 Street, 04-628 Warsaw, Poland; 5 Department of Coronary Artery Disease and Structural Heart Disease, Institute of Cardiology, Alpejska 42 Street, 04-628 Warsaw, Poland; 6 Department of Intensive Cardiac Care, Institute of Cardiology, Alpejska 42 Street, 04-628 Warsaw, Poland

**Keywords:** Transcatheter aortic valve implantation, Vascular complications, Long-term mortality

## Abstract

Vascular complications are the main safety limitations of transcatheter aortic valve implantation (TAVI). The aim of the study was to assess the incidents, predictors, and the impact of early vascular complications on prognosis after TAVI. This was a single-center analysis of vascular complications related to TAVI. Early vascular complications were defined as incidents within 30 days after TAVI and comprised complications related to transvascular: transfemoral/transsubclavian ,and transapical bioprosthesis implantation. Evaluated risk factors were: (1) clinical characteristics, (2) TAVI route, and (3) center experience. In patients with transvascular TAVI the impact of: (1) diameters of access arteries, vascular sheathes and difference between them, (2) arterial wall calcification, and (3) ProStar devices used for access site closure were assessed. Arterial wall calcification and arteries diameters were measured by 64-slice computer tomography. Arterial wall calcification was graded according to 5° scale. Results: between 2009–2011; follow-up 1–23 months (12 ± 15.55), 83 consecutive patients, and 62–91 (81.10 ± 7.20) years, underwent TAVI: 67 (80.72 %) patients had transvascular, and 16 (19.27 %) patients had transapical bioprosthesis implantation. We noted 44 (53.01 %) early vascular complications: 17 (20.48 %) were major and 27 (32.53 %) were minor incidents. Independent predictors of early vascular complications were: history of anaemia (OR 3.497: 95 % CI [1.276–9.581]; *p* = 0.014), diabetes (OR 0.323: 95 % CI [0.108–0.962]; *p* = 0.042), percutaneous coronary intervention performed as preparation for TAVI (OR 4.809: 95 % CI [1.172–19.736]; *p* = 0.029), and arterial wall calcification (OR 1.945: 95 % CI [1.063–3.558]; *p* = 0.03). Of 6 (7.22 %) in-hospital and 10 (12.98 %) late deaths: 5 (83.33 %) patients and 8 (80 %) patients respectively had post-procedural vascular complications. Vascular complications, which occurred in 30-days after TAVI, predict late mortality (*p* = 0.036). Conclusions derived were: (1) TAVI patients with history of anaemia and diabetes required careful monitoring for early vascular complications. (2) If coronary intervention before TAVI is required, it should be performed in the time allowing vascular injuries to heal. (3) Calcification of access arteries is an independent predictor of post-procedural vascular complications; therefore, its estimation should be a regular element of preceding computer tomography. (4) Vascular complications seem to be predictors of late mortality after TAVI.

## Introduction

Transcatheter aortic valve implantation (TAVI) is dedicated for inoperable or high surgical-risk patients with severe aortic stenosis [1, 2]. Although the feasibility and efficacy of TAVI has been proved, the procedure is still related to inherent complications [2–10]. Vascular complications (VCs) are one of the most common early adverse events after TAVI, which affect from 9.5 % to even more than 50 % of TAVI populations [3]. The majority of researchers assumed that, a high percentage of VCs are directly associated with technical aspects and complexity of the procedure [5–9]. On the other hand, a few reports suggest that despite down-sizing the sheath diameters and device sizes, with increasing operators’ experience, the prevalence of VCs still remains at a relatively high level [5, 6]. Although TAVI population is a special group of elderly with many comorbidities, which may be crucial for constantly high proportion of VCs, the impact of patients’ characteristics on early VCs has not been sufficiently assessed so far.

Early VCs are usually associated with serious bleeding and often require urgent surgical or invasive treatment and blood transfusions. Therefore, VCs are known as the most frequent causes of in-hospital mortality and seem to be the main safety limitation of this procedure [3–9]. The importance of the issue has additionally increased since the long-term results of PARTNER trial confirmed that early VCs worsen late survival [4].

In the face of data mentioned above, the main objectives of our study was to evaluate the incidents of early VCs and to define their predictors resulting from arterial morphology, technical aspects of the procedure and patients’ clinical characteristics. Furthermore, we assessed the impact of early VCs on post-TAVI prognosis.

## Materials and methods

### Patients population and study design

It was a single-center, observational study of early VCs related to TAVI. We collected the data of consecutive patients who underwent TAVI at our center between January 2009 and October 2011. The analysis was retrospective until February 2011 and from that point on it became a prospective one. VCs were defined by the updated Valve Academic Research Consortium (VARC)-2 scale [11]. Patients were qualified for TAVI by our Heart Team when they met the criteria approved by the ESC/European Association of Cardio-Thoracic Surgeons (EACTS) in collaboration with the European Association on Percutaneous Coronary Interventions (EAPCI) in 2008 [12] or when there were no technical possibilities to perform classic surgery. The inclusion criteria were: severe symptomatic aortic valve stenosis with a valve area ≤1.0 cm^2^, logistic European System for Cardiac Operative Risk Evaluation (EuroSCORE) ≥20 %, or logistic EuroSCORE <20 % with concomitant condition, which was contraindication for classic surgery (e.g. advanced osteoporosis or porcelain aorta). The inclusion criteria remained unchanged throughout the study.

The procedure was performed via transvascular approach: transfemoral (TF-) and transsubclavian (Tsc-); or transapical (TA-AVI). TA-AVI was performed when transvascular procedure was not possible.

Major and minor VCs were assessed together because of the small number of participants. Early VCs were defined as incidents within 30 days after TAVI and comprised complications related to TF/Tsc-AVI and TA-AVI. The prognostic value of risk factors resulting from clinical characteristics, preceding invasive procedures, route of bioprosthesis implantation, and center experience was assessed in all participants.

In the subset of patients with transvascular TAVI, we evaluated the impact of the following arterial morphology and technical aspects of the procedure: (1) minimal internal diameter (MID) of the access artery, (2) arterial wall calcification (CALC), (3) sheath external diameter (SED), (4) difference between MID and SED, (5) ProStar devices used for access site closure, (6) bioprosthesis type, and (7) TF-/Tsc- access site.

The study group was divided into two subgroups to assess the influence of a learning curve on early VCs. Subgroups presented consecutive patients that underwent TAVI earlier and later during the follow-up period.

### Technical aspects of the procedure

Two types of prostheses were implanted: Edwards-Sapien (ES) bioprosthesis of 23 and 26 mm diameter or Sapien XT (SXT) bioprostheses (Edwards Lifesciences) of 23, 26 and 29 mm diameter, and CoreValve (CV) bioprosthesis (CoreValve Revalving System, Medtronic) of 26 and 29 mm diameter. The prosthesis implantation was accomplished with one of the corresponding delivery systems: Retroflex 3 vascular sheath system designed for ES with 28-Fr (9.2 mm) or 25-Fr (8.4 mm) outer diameters, the Novaflex system used for SXT with 21.7-Fr (7.16 mm) or 22.5-Fr (7.5 mm) outer diameters and the Medtronic CoreValve Accutrak Delivery Catheter System for CV—21.8-Fr (7.19 mm). For TA-AVI Ascendra and Ascendra 2 systems were used with 24-Fr and 26-Fr (Edwards Lifesciences) sheaths. Transfemoral or transsubclavian access was used for the CV prosthesis. For the ES and SXT prostheses transfemoral or transapical access was applied. The type of bioprosthesis and the route of implantation were adjusted to the size of the native valve annulus and to the diameter and status of the peripheral arteries, based on the computed tomography angiography and transesophageal echocardiography (TEE) findings.

The vascular access in the majority of TF-AVI was achieved percutaneously by pre-closure technique with the use of ProStar XL™ (Abbott Vascular Laboratories Inc.), a suture based system, or by a surgical arterial cut-down. In Tsc-AVI arterial access was always surgical. After prosthesis deployment, arteries were sutured surgically or by using 10-Fr ProStar device.

Pre-closure technique was based on the puncture of the common femoral artery defined by contralateral ilio–femoral angiography. A single ProStar device was placed in the femoral artery before the aortic valve deployment. After prosthesis implantation, before the femoral artery closure with ProStar sutures, in the ipsilateral external iliac artery, a properly sized balloon was placed proximally to the sutures from the contralateral femoral artery and then inflated. After tightening the sutures, the balloon was deflated and haemostasis confirmed. Afterwards, a final ilio–femoral angiogram was performed from the contralateral side.

### Vascular imaging

The route of TAVI was chosen on the basis of computed tomography angiography (CTA) examination, performed with Dual Source CT (Somatom Definition, Siemens).

CTA was routinely performed; the following parameters were evaluated: the diameter and calcification of the aorta, ilio–femoral, subclavian arteries, and the aortic annulus, the distance between the aortic annulus plane and the ostia of the coronary arteries, and the morphology of the aortic valve. The retrospective ECG-gated CTA was performed to assess the aortic root and the ascending aorta, followed by non ECG-gated CTA of the abdominal aorta and ilio–femoral arteries to the proximal part of the femoral common arteries.

The diameters of the common femoral arteries were measured below the inguinal ligament in the axial projection (perpendicular to the long axis of the vessels). The diameters of the subclavian arteries were assessed in transverse projection along their course. The real vessel diameter was defined as the distance between the internal vessel walls. However, in the case of wall calcification the real diameter was the vessel lumen—the internal diameter was reduced by the calcification dimension defined as the minimal internal diameter (MID). The distribution and extent of arterial wall calcification (CALC) were evaluated by the semi-quantitative method and graded according to a 5 ° scale as: 0—without CALC, 1—CALC < 25 %, 2—CALC 25–50 %, 3—CALC 50–75 %, 4—CALC > 75 % of arterial perimeter.

The study was approved by the local Ethics Committee and a written informed consent was obtained from each patient in the prospective part of the study.

### Statistical analysis

Uni-and multivariate statistical analyses were performed by means of the SAS system. Categorical data were expressed as frequency and percentages; continuous variables were presented as mean ± SD. Comparisons were made with the χ^2^ test or the Fisher exact test for categorical variables and with the nonparametric Wilcoxon test for continuous variables. The multivariate analysis was performed with a stepwise logistic regression analysis with estimate odds ratio (OR) and 95 % confidence interval (CI) to identify independent variables predicting the risk for early VCs. The impact of early VCs on mortality after TAVI was assessed with a Cox regression analysis with estimated hazard ratio (HR) and 95 % CI and log-rank test with Kaplan–Meier curves. Due to the small number of noted late deaths the impact of VCs on mortality was primarily assessed with adjustment for each defined predictor of VCs separately and subsequently including all predictors. For all statistical tests, a significance level of *p* < 0.05 was used, which also was the *p* level for entry in the multivariate analysis.

## Results

The study included 83 consecutive patients (pts). Patients’ characteristic is presented in Table 1.

**Table 1:** Baseline characteristics of 83 patients (pts)

Age-yr mean ± SD	81.10 ± 7.20
>85-yr—no. (%)	31 (37.34)
Female sex—no. (%)	54 (65.06)
Logistic EuroSCORE % (mean ± SD)	2.86–59.01 (24.96 ± 12.71)
Implantation	
TF-AVI/Tsc-AVI—no. (%)	59/8 (71.08/9.64)
TA-AVI—no. (%)	16 (19.28)
NYHA class—no. (%)	
II	16 (19.28)
III	52 (62.65)
IV	15 (18.07)
Coronary artery disease—no.(%)	62 (74.69)
Previous myocardial infarction—no. (%)	20 (24.09)
Previous coronary intervention—no. (%)	38 (45.78)
PCI	26 (31.33)
≤6 months pre-TAVI	18 (21.68)
CABG	12 (14.46)
COPD—no. (%)	25 (30.12)
Atrial fibrillation—no. (%)	32 (38.55)
Permanent pacemaker—pre/post TAVI—no. (%)	12/26 (14.45/31.32)
Pulmonary hypertension—no. (%)	47 (56.63)
Extensively calcified aorta—no. (%)	7 (8.43)
Osteoporosis—no. (%)	23 (27.71)
BMI (kg/m^2^) mean ± SD	25.54 ± 4.0

*BMI* body mass index, *CABG* coronary–artery bypass grafting, *COPD* chronic obstructive pulmonary disease, *NYHA* New York Heart Association, *TA-AVI* transapical aortic-valve implantation, *TF-AVI* transfemoral aortic valve implantation, *Tsc-AVI* transsubclavian aortic valve implantation, *PCI* percutaneous coronary intervention

In 67 (80.72 %) pts, TAVI was performed from the vascular approach: 59 (71.08 %) pts had TF-AVI and 8 (9.64 %) pts had Tsc-AVI. TA-AVI was performed in 16 (19.28 %) pts. ES and SXT bioprostheses were used in 37 (44.57 %) and 3 (3.61 %) pts, respectively. CV bioprosthesis was implanted in 43 (51.8 %) pts.

### Vascular complications

Forty four (53.01 %) pts experienced post-procedural VCs. Major VCs occurred in 17 (20.48 %) pts, while 27 (32.53 %) pts had minor VCs.

In TF-/Tsc-AVI group, 35 (52.23 %) pts experienced VCs; 13 (37.14 %) pts had major complications. In TA-AVI group, 9 (56.25 %) pts had VCs; major VCs were noted in 4 (25 %) pts. Minor VCs occurred in 22 (62.82 %) TF-/Tsc-AVI and 5 (31.25 %) TA-AVI pts respectively (Table 2).

**Table 2:** VCs and in-hospital deaths in 83 pts after TAVI

Type of VCs	All VCs, n = 44	Major VCs, n = 17	Minor VCs, n = 27	Death n = 3
Aorta				
Rupture	1^a^	1^a^	–	1^a^
Dissection	1^b^	1^b^	–	1^b^
Retroperitoneal bleeding	3^c^	3^c^	–	
Femoral artery				
Rupture	3^c^	3^c^	–	1^c^
Dissection	3	2	1	–
Occlusion/stenosis	2^d^	–	2^d^	–
Arterio-venous fistula	1	–	1	–
Hematoma/bleeding	8	1	7	–
ProStar XL failure	10^d^	1	9^d^	–
Femoral stent dislocation	1	1	–	–
Distal embolization resulting in				
Amputation	1	1	–	–
Embolectomy	1	–	1	–
Subclavian artery				
Perforation	1	1	–	–
Dissection	1^b^	1^b^	–	–
Hematoma/bleeding	4	2	2	–
Connected with TA-AVI				
Pleural bleeding/hematoma	6	2	4	–
Subcutaneous hematoma	1	–	1	–
Interstinal haematoma	1	1	–	–
Intervention				
Balloon angioplasty	7	1	6	–
Femoral stenting	4	2	2	–
Vascular surgery	9	5	4	–
Retoracotomy	4	4	–	2
Blood transfusion	39	17	22	3

*TA-AVI* transapical aortic valve implantation

^a^pt after TA-AVI

^b^pt with aorta and subclavian artery dissection with pericardial tamponade

^c^pts with parallel femoral artery rupture and retroperitoneal bleeding

^d^pt with artery stenosis resulting from ProStar failure

### Predictors of early VCs

We assessed the impact of patients’ characteristics, preceding interventions, and the approach to a bioprosthesis implantation on early VCs. In uni-and multivariate analyses the history of anemia, percutaneous coronary intervention (PCI) performed as preparation for TAVI during preceding 6 months, and diabetes were independent predictors of early VCs (Table 3).

**Table 3:** Impact of clinical characteristics on early VCs after TAVI in 83 patients—uni-and multivariate analyses

Risk factors	VCs no. (%)		*p* value	OR [95 % CI]; *p* value
	Yes (n = 44)	No (n = 39)		
Hypertension	34 (77.27)	34 (87.17)	0.24	–
Renal failure^a^	25 (56.81)	24 (61.53)	0.66	–
History of bleeding	6 (13.63)	6 (15.38)	0.82	–
History of anemia^b^	29 (65.90)	14 (35.89)	0.006	3.497 [1.276–9.581];0.014
Age-yr mean ± SD	81.45 ± 7.83	80.75 ± 6.58	0.21	–
Age >85-yr	18 (40.90)	9 (35.89)	0.059	–
Female sex	29 (65.90)	25 (64.10)	0.86	–
Stroke/TIA	3 (6.81)	10 (25.64)	0.018	–
Diabetes mellitus	12 (27.27)	21 (53.84)	0.013	0.323 [0.108–0.962];0.042
Implantation				
TF-AVI + Tsc-AVI/TA-AVI	29 + 6 (79.54)/9 (20.45)	30 + 2 (82.05 %)/7 (17.94)	0.37	–
BMI (kg/m^2^) < 25.54 ± 4.0	30 (68.18)	11 (28.20)	0.46	–
PCI ≤ 6 months before TAVI	14 (31.81)	4 (10.25)	0.01	4.809 [1.172–19.736];0.029

*BMI* body mass index, *PCI* percutaneous coronary intervention, *TA-AVI* transapical aortic valve implantation, *TF-AVI* transfemoral aortic valve implantation, *TIA* transient ischemic attack, *Tsc-AVI* transsubclavian aortic valve implantation

^a^Serum creatinine ≥200 μmol/L or GFR <60 ml/min/1.73 m^2^

^b^History of anemia and/or hemoglobin <12.0 g/dL day before TAVI

In a subset of 67 patients who underwent TF-AVI/Tsc-AVI we evaluated the impact of femoral and subclavian arterial morphology and technical aspects of the procedure on early VCs. The procedural and anatomical characteristics of the study population are presented in Table 4. The impact of the assessed risk factors on early VCs after transvascular TAVI is presented in Table 5.

**Table 4:** Procedural and anatomical characteristics of 67 study pts with transvascular TAVI

	VCs	
	Yes (n = 35^a^)	No (n = 32)
Arterial wall calcification (CALC)^b^—no. (%)		
0	7 (20.58)	16 (50)
I	16 (47.05)	10 (31.25)
II	8 (23.52)	5 (15.62)
III	3 (8.82)	1 (3.12)
CALC^b^ mean ± SD	1.2 ± 0.88	0.71 ± 0.85
Outer sheath size (Fr)—no. (%)		
21.8	25 (71.42)	23 (71.87)
21.7	1 (2.85)	–
22.5	1 (2.85)	1 (3.12)
25	3 (8.57)	6 (18.75)
28	5 (14.28)	2 (6.25)
TF-/Tsc-AVI—no. (%)	29 (82.85)/6 (17.14)	30 (93.75)/2 (6.25)
CV/ES and SXT—no. (%)	22 (62.85)/13 (37.14)	21 (65.625)/11 (34.37)
ProStar—no. (%)	23 (65.71)	23 (71.81)
Minimal internal diameter (MID) (mm) mean ± SD	8.02 ± 1.30	8.78 ± 1.54
Sheath external diameter (SED) (mm) mean ± SD	7.70 ± 0.77	7.63 ± 0.74
Difference between MID and SED (MID–SED) (mm) mean ± SD	0.31 ± 1.42	1.14 ± 1.59

*CALC* arterial wall calcification, *CV* CoreValve prosthesis, *ES* Edwards Sapien protsthesis, *Fr* French, *SXT* Sapien XT prosthesis, *TF-AVI* transfemoral aortic valve implantation, *Tsc-AVI* transsubclavian aortic valve implantation

^a^1 pt excluded from MID, MID–SED, CALC assessment due to lack of preceding CTA

^b^CALC was graded according to 5° scale: 0—no CALC, I—CALC 0–25 %, II—CALC 25–50 %, III—CALC of 50–75 %, IV—CALC > 75 % of arterial wall perimeter

**Table 5:** Impact of procedural and anatomical characteristics on early VCs in 67 patients with transvascular TAVI—univariate analysis

Risk factors	VCs		*p* value	OR [95 % CI]; *p* value
	Yes (n = 35^a^)	No (n = 32)		
MID (mm) mean ± SD	8.02 ± 1.30	8.78 ± 1.54	0.035	0.675 [0.463–0.984]; 0.04
SED (mm) mean ± SD	7.70 ± 0.77	7.63 ± 0.74	1.0	0.933 [0.433–2.011]; 0.85
MID–SED (mm) mean ± SD	0.31 ± 1.42	1.14 ± 1.59	0.017	0.643 [0.425–0.972]; 0.03
CALC mean ± SD	1.2 ± 0.88	0.71 ± 0.85	0.02	1.945 [1.063–3.558]; 0.03^b^
ProStar—no. (%)	23 (65.71)	23 (71.81)	0.58	–
TF-/Tsc-AVI—no. (%)	29 (82.85)/6 (17.14)	30 (93.75)/2 (6.25)	0.16	–
CV/ES and SXT—no. (%)	22 (62.85)/13 (37.14)	21 (65.625)/11 (34.37)	0.74	–

*CALC* arterial wall calcification, *CV* CoreValve prosthesis, *ES* Edwards Sapien protsthesis, *MID* minimal internal diameter, *SED* sheath external diameter, *SXT* Sapien XT prosthesis, *TF-AVI* transfemoral aortic valve implantation, *Tsc-AVI* transsubclavian aortic valve implantation

^a^1 pt excluded from MID, MID–SED, CALC assessment due to lack of preceding CTA

^b^for CALC graded according to 5°  scale

In a multivariate stepwise analysis, CALC was an independent predictor of early VCs (OR 1.945: 95 % CI [1.063–3.558]; *p* = 0.03).

### Impact of a center experience on early VCs

In the first 42 (50.6 %) pts we noted 19 (45.23 %) early VCs, subsequently 19 (46.34 %) VCs were noted in 41 (49.39 %) pts who underwent TAVI later. The learning curves had no impact on early VCs after TAVI (*p* = 0.09).

### Impact of early VCs on early and late mortality after TAVI

During 30 days after TAVI 6 (7.22 %) pts died; 5 (83.33 %) of them had VCs. Post-procedural VCs were the cause of 3 early deaths (Table 2). The other 3 pts died due to: 1—CHF exacerbation, 2—SCD. In the univariate Cox regression analysis VCs did not increase significantly the risk of early mortality (*p* = 0.31); (HR 1.812: 95 % CI [0.332–9.894]; *p* = 0.49).

**Fig. 1:**
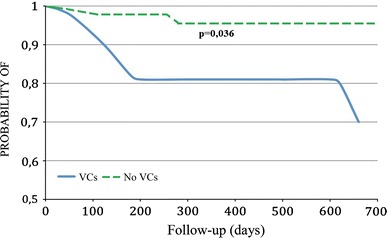
Impact of early VCs on long-term prognosis after TAVI with Kaplan–Meier curves in 75 pts

A long-term follow-up included initially 77 (92.77 %) pts discharged from the hospital; 2 pts were lost in follow-up. The final follow-up 1–23 months (12 ± 15.55) included 75 participants. We noted 10 (12.98 %) late deaths: 6—sudden deaths during sleep, 3—CHF exacerbation, 1—malignant neoplastic disease. Post-procedural VCs occurred in 8 (80 %) of these pts. In a univariate analysis, early VCs had no impact on late mortality (*p* = 0.057) (HR: 4.229 95 % CI [0.894–20.016]; *p* = 0.069). In the log-rank test, early VCs significantly increased late mortality (*p* = 0.036) (Fig. 1). The impact of VCs on late mortality remained significant only after adjustment for diabetes (*p* = 0.04) (HR 5.70: 95 % CI [1.149–28.280]; *p* = 0.03) (Table 6).

**Table 6:** Impact of early VCs on late mortality in univariate analysis and after adjustment for predictors of VCs

Impact of VCs	HR [95 % CI]; *p* value
Univariate analysis	4.229 [0.894–20.016]; 0.06
Adjustment for	
Diabetes mellitus	5.70 [1.149–28.280]; 0.03
History of anemia	3.20 [0.642–15.983]; 0.15
PCI ≤ 6 months before TAVI	3.638 [0.739–17.920]; 0.11
CALC	3.515 [0.690–17.909]; 0.13
All predictors of VCs	2.940 [0.412–21.002]; 0.28

*CALC* arterial wall calcification, *PCI* percutaneous coronary intervention

## Discussion

Vascular complications and bleeding related with them has always been the main cause of morbidity and mortality after percutaneous interventions [13]. Although TAVI is considered to be a minimally invasive procedure, post-procedural VCs are major disadvantages of this method [3–10]. In spite of the fact that the proportion of early VCs affects from nearly 10 % to more than 50 % of TAVI, only few previous reports were dedicated to these complications [5–10].

On the basis of one-center experience we showed that early VCs occurred in 53 % of the patients. In respect to other studies [5–10] the reported ratio of VCs is high but is consistent with the one observed by Mussardo et al. who also described one-center TAVI experience [5]. The majority of VCs noted in our analysis were caused by minor complications, which comprised more than 60 % of them. Minor VCs mainly resulted from ProStar device failures and hematomas/bleeding at the access site. Similarly, Toggweiler or Hayashida observed that the proportion of early VCs was mainly amplified by a high number of minor VCs [7, 8]. Furthermore, Gurvitch and Genereux suggested that the relation mentioned above results from the mere VARC definition, which is very comprehensive [3, 9]. In accordance with VARC-2 scale, post-procedural hematomas at the acess site can be considered as minor VCs [11]. Moreover, novel VCs definition apart from criteria of post-procedural haemoglobin drop still considered parallel blood transfusion criteria. Although VARC-2 scale pointed out that: ‘bleeding complication has to be the result of overt bleeding and cannot be adjudicated based on blood transfusions alone’, it does not determine post-procedural haemoglobin level in which RBC transfusion should be considered [11]. As a result, all incidents of transfusion and the number of transfused RBC units due to VCs are based on an individual physician’s decision and can increase the rate of major VCs. It should be noted that elderly people especially those ones with coexisting anemia might be very sensitive for even a small blood loss. Thus proposed VCs definition requires further observation rather than criticism.

Although a few studies demonstrated a direct impact of technical aspects and arterial morphology on post-procedural VCs [7–9], other reports proved that a significant reduction of early VCs was not obtained despite an improvement in the procedure and a reduction in the dimensions of devices and sheaths [5, 6]. The constant proportion of early VCs could result from other not investigated factors such as clinical characteristics.

Our study is the first one, which demonstrates that the history of anemia, preceding PCI and diabetes were independent predictors of early VCs.

Only few previous analyses indicated the impact of patients’ characteristics on post-procedural VCs. Female sex was found to be a predictor of major VCs [5, 9, 14]. Other investigators reported that early VCs occurred more often in patients with peripheral arterial disease [8, 9, 15].

Chronic anemia occurs commonly in elderly population. The impact of anemia on early VCs after TAVI has not been evaluated yet; however, Halliday and co-workers on the basis of one-center retrospective study proved that the history of anemia increased the risk for serious bleeding directly caused by early VCs [16]. Furthermore, anemia is a commonly known risk factor of bleeding after coronary interventions [17]. On the other hand, this outcome may reflect only the impact of pre-procedural anemia on a higher number of post-TAVI transfusions that wrongly amplify the number of VCs.

Diabetes is a known predictor of bleeding complications [18]; however, there are limited data on how it impacts TAVI population. Only Halliday et al. and Pilgrim and colleagues demonstrated that diabetes increased significantly the risk for major and life-threatening TAVI bleeding [16, 19].

Although 40–60 % of TAVI patients required preceding coronary interventions [20–22], the impact of preceding PCI on early VCs, performed as preparation for TAVI, has not been the subject of a separate analysis so far. Similarly to our results, Dewey et al. on the basis of two-center analysis proved that concomitant CAD, defined as preceding CABG or PCI, increased the risk for in-hospital VCs and major bleeding after TAVI [20]. Contrary, Gasparetto et al. on the basis of one-center prospective registry, showed that preceding coronary intervention seems to be safe [22].

The influence of diabetes and PCI on early VCs remains inscrutable. Diabetes affects vascular adverse remodeling, increases arterial wall calcification, and may be a predictor of peripheral arterial disease (PAD); thus, it enhances the risk for early VCs [15]. PCI can increase the risk for VCs due to mechanical injuries of the access vessels through which TAVI is to be performed shortly.

With regard to procedural characteristics and arterial morphology, we proved that a greater arterial wall calcification, small arterial diameters and small difference between arterial and sheath diameters, significantly increased the risk for early VCs. Moreover, we found that arterial wall calcification was an independent predictor of early VCs. Each degree of extensive calcification almost doubled the risk for VCs. These results are consistent with other reports [5, 7, 8]. Hayashida and co-authors in a retrospective analysis of 127 pts, proved that wall calcification was an independent predictor of early VCs [7]. Similarly, it was proved that the small ratio between sheath and arterial diameters increased the risk for early VCs [5, 7]. Furthermore, Hayashida et al. showed that the value of this ratio > 1.5 is an independent predictor of early VCs, and of in-hospital and 30-day mortality [7]. These findings suggest that the assessment of diameters of the access site vessels, similarly to arterial calcification in preceding CTA, should be a routine part of patients’ qualification. Furthermore, there is a need for a standardized scale for CTA arterial calcification.

Only 3 of our patients had a new SXT prosthesis implanted during the study; therefore, we could not assess the impact of the newer—NovaFlex delivery systems with smaller sheath sizes on post-procedural VCs. However, similarly to Hayashida et al., we found that the use of ES bioprostheses with larger sheath diameters vs CV bioprostheses with small 18-Fr vascular systems had no impact on early VCs, which most probably results from the small number of patients studied [7].

Subsequently, we showed that the use of the percutaneous closure technique had no impact on VCs and seems to be safe. Although van Mieghem et al. proved that it was the main cause of major VCs [9] our finding has been confirmed by other reports, which clearly established the efficacy and safety of access site closure devices [23, 24].

Our analysis showed that operators’ experience had no impact on early VCs. This is contrary to other studies, which proved that the proportion of post-procedural VCs, bleeding and blood transfusions decreased significantly over years due to growing center experience [7, 9, 25]. Of note, Lange et al. on the basis of consecutively included 412 TAVI participants noticed the reduction in TAVI complications only after 300 initial cases, which according to authors attests to the complexity of the procedure [25]. Thus, our outome may be biased by small number of patient operated per year and the prolonged learning curve.

We described our 2-year experience from the beginning of using TAVI, when the procedures were performed under proctor control until self-dependent implantations. Therefore, our outcomes are of a particular value. They confirm that the most important risk factors of VCs results from patients’ clinical and anatomical characteristics.

Finally, we did not prove the impact of VCs on early mortality probably due to the small number of post-proceudral deaths. However, the crucial is the finding that the early VCs can affect long-term prognosis. Although covariates such as diabetes, anemia, and arterial wall calcification invalidated this relation, the result was based only on 10 events. Thus, the impact of VCs on survival may be relevant for real-life large TAVI population. Few reports assessed the impact of VCs on long-term survival [4, 26, 27]. SOURCE or Canadian registries showed a significant correlation between early VCs and one-year mortality [26, 27]. Two-year outcomes of PARTNER A study proved that major post-procedural VCs increased late mortality by more than 1.5-times [4]. Unfortunately, the underlying cause of the relationship between early VCs and late mortality after TAVI has never been investigated so far. It should be taken into account that although noted late deaths were not directly related to VCs, 60 % of them occurred during sleep and cardio-vascular events are probable causes of them. As long as PAD is a known risk factor for cardio-vascular events including death, early VCs may be an exponent of advance, generalized atherosclerosis. This is particularly true in case of TAVI population—a group of patients with high prevalence of PAD. This hypothesis is supported by recent German Registry results. On the basis of 1,315 patients, Sinning et al. proved that PAD not only increased the risk of VCs, but also was an independent predictor of mortality after TAVI [15].

Although our results require further investigations, they could be considered as guidelines for peri-procedural TAVI care. In terms of early VCs, patients with pre-procedural anemia require intensive monitoring in the early post-TAVI period. In patients with concomitant diabetes, not only intensive post-TAVI monitoring is required, but also careful assessment of vascular access in CTA examination. If PCI before TAVI is necessary, it should be performed in the time allowing vascular injuries to heal or should be performed only by radial access or parallelly with TAVI. Finally, advanced arterial wall calcification and small diameter of access vessels should determine the consideration of alternative rather than transvascular approaches.

### Study limitation

The small number of participants and partially retrospective observation were the obvious limitations of the presented study. Because of the small sample size we could not determine independent risk factors separately for major VCs. We could not fully estimate the impact of learning curve on VCs or impact of VCs on mortality for the same reason.

Moreover, the presented study described the early experience of our center; therefore, we assessed the impact of 22- and 24-Fr Edwards Sapien sheaths of the RetroFlex delivery system, which are no longer commercially available in Europe, instead of the NovaFlex system used currently.

## Conclusions

1. TAVI patients with history of anemia or diabetes required careful monitoring for VCs in the early post-TAVI period.
2. If coronary intervention before TAVI is required, it should be performed in the time allowing vascular injuries to heal.
3. Small arterial diameters and small difference between arterial and sheath diameters increased the risk for early VCs.
4. Calcification of access arteries is a significant predictor of early VCs; therefore, its estimation should be a regular element of preceding CTA.
5. A specific scale for arterial calcification assessment is required.
6. Early VCs seems to be predictors of late mortality in TAVI population. However, this finding requires further researches.
